# A novel role for the rat retrosplenial cortex in cognitive control

**DOI:** 10.1101/lm.032136.113

**Published:** 2014-02

**Authors:** Andrew J.D. Nelson, Emma L. Hindley, Josephine E. Haddon, Seralynne D. Vann, John P. Aggleton

**Affiliations:** 1 School of Psychology, Cardiff University, Cardiff CF10 3AT, United Kingdom; 2 Institute of Psychological Medicine and Clinical Neurosciences, Cardiff University, Cardiff CF14 4XN, United Kingdom

## Abstract

By virtue of its frontal and hippocampal connections, the retrosplenial cortex is uniquely placed to support cognition. Here, we tested whether the retrosplenial cortex is required for frontal tasks analogous to the Stroop Test, i.e., for the ability to select between conflicting responses and inhibit responding to task-irrelevant cues. Rats first acquired two instrumental conditional discriminations, one auditory and one visual, set in two distinct contexts. As a result, rats were rewarded for pressing either the right or left lever when a particular auditory or visual signal was present. In extinction, rats received compound stimuli that either comprised the auditory and visual elements that signaled the same lever response (congruent) or signaled different lever responses (incongruent) during training. On conflict (incongruent) trials, lever selection by sham-operated animals followed the stimulus element that had previously been trained in that same test context, whereas animals with retrosplenial cortex lesions failed to disambiguate the conflicting response cues. Subsequent experiments demonstrated that this abnormality on conflict trials was not due to a failure in distinguishing the contexts. Rather, these data reveal the selective involvement of the rat retrosplenial cortex in response conflict, and so extend the frontal system underlying cognitive control.

Throughout life we must select between conflicting responses. Increased demands on cognitive control occur when multiple responses may be appropriate, when in the presence of ambiguous stimuli, or when a dominant but task-inappropriate response should be suppressed. The Stroop Test (Stroop 1935) embodies the problems of cognitive control in the presence of conflicting responses. The Stroop task requires participants to read a word or name the color of the ink with which the word is written. Color–word combinations comprise either congruent (e.g., the word “red” in red ink) or incongruent word and ink combinations (e.g., the word “red” written in blue ink) and participants must use the task-relevant attribute of the compound (e.g., name the word) to control responding while ignoring the irrelevant attribute (e.g., ignore the color of the ink). Use of the Stroop Test, along with other tests of cognitive control, has consistently highlighted the importance of frontal regions for the detection and resolution of response conflict. More specifically, both studies of patients with brain injury and functional MRI findings have repeatedly implicated the anterior cingulate cortex (ACC) and dorsolateral prefrontal cortex (DLPFC) (e.g., MacDonald et al. 2000; Rushworth et al. 2004, 2012; Carter and van Veen 2007). These frontal regions cannot function in isolation, and evidence that they might require interactions with posterior cingulate areas comes from the recent finding of significant correlations between Stroop Test performance and variations in the microstructure of the left cingulum bundle, as revealed by diffusion MRI (Metzler-Baddeley et al. 2012). This association was particularly robust for that part of the bundle adjacent to the retrosplenial cortex (areas 29, 30), a cortical region reciprocally interconnected with the ACC and DLPFC via the cingulum (Mufson and Pandya 1984; Morris et al. 1999).

Although the retrosplenial cortex is important for a range of spatial and mnemonic functions (Maguire 2001; Vann et al. 2009), the impact of retrosplenial damage upon decision making and response conflict has received scant attention. Although an early study reported deficits on the Wisconsin Card Sorting Test in a patient with unilateral pathology involving the retrosplenial cortex (Valenstein et al. 1987), there have been few follow-up studies of cognitive control. A persistent problem concerns the difficulty of locating any cases with confirmed, selective retrosplenial pathology (Maguire 2001; Vann et al. 2009), highlighting the particular value of comparative lesion studies of this region.

For this reason, rats with excitotoxic lesions of the retrosplenial cortex were tested on a rodent analog of the Stroop Test (Haddon and Killcross 2005, 2006a,b; Haddon et al. 2008). This behavioral task, which examines choice behavior under conditions of cue and response conflict, depends on the integrity of the rat medial prefrontal cortex and ACC (Haddon and Killcross 2006a), cortical regions analogous to those implicated in human studies of the Stroop task. In this task, rats concurrently acquire two conditional discriminations, one visual and one auditory, in two distinct contexts. Consequently, each rat acquires four distinct instrumental contingencies (Fig. 1). At test, animals receive compound audiovisual stimuli either composed of those stimulus elements that had elicited the same response (“congruent” trials) or different responses (“incongruent” trials) during training. Responses during incongruent stimulus compounds are defined as correct or incorrect according to whether they are appropriate to the test context. Thus, rats must rely on contextual information to disambiguate conflicting response information in a manner analogous to the use of task-setting instructions to identify the task-relevant attribute in the human Stroop paradigm (see Haddon and Killcross 2007). If the retrosplenial cortex is critical for resolving response conflict, lesions of this area should selectively disrupt performance on incongruent trials when animals are required to choose between competing responses. As a failure to learn or process contextual information could, in principle, contribute to any retrosplenial lesion impairment on this task, we also assessed the ability of animals with retrosplenial cortex lesions to use contextual information to guide instrumental behavior. Subsequently, we explored the nature of the representations underpinning performance on this task by examining the impact of motivational manipulations on response conflict performance.

**Figure 1.**
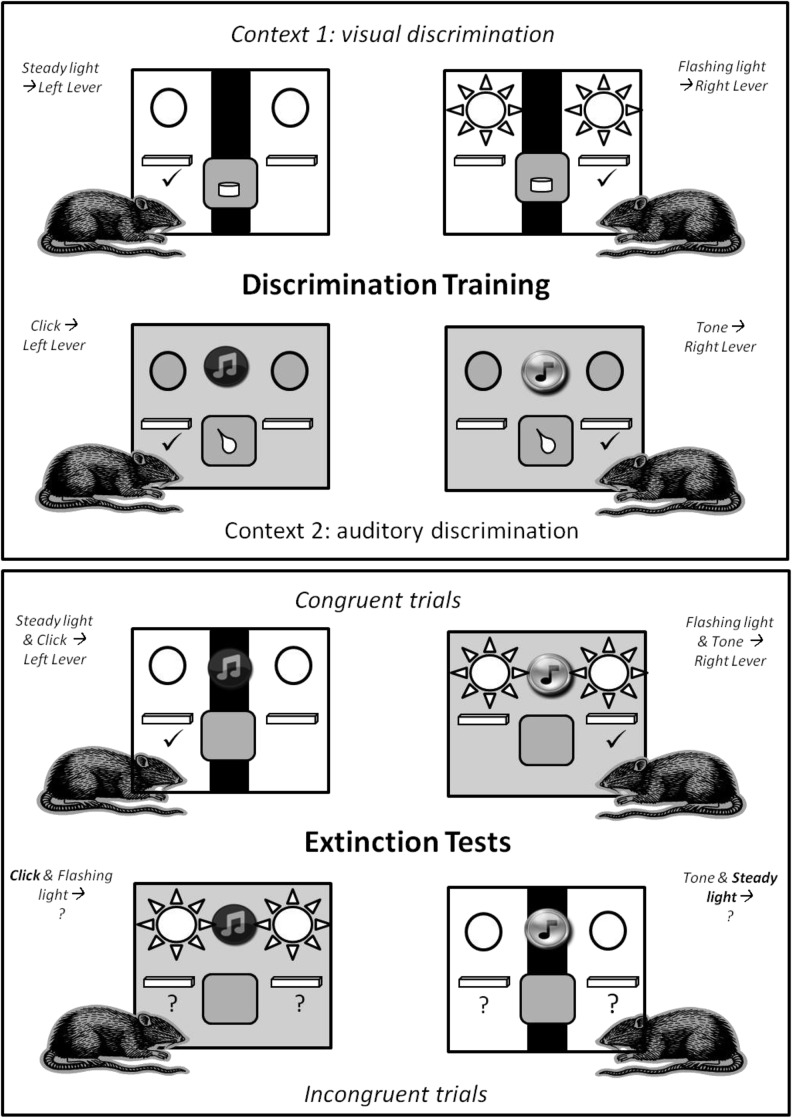
Experimental design. Animals acquired two conditional discriminations (one auditory and one visual) in two distinct contexts with different rewards (food pellets and sucrose solution). During extinction tests, animals received audiovisual compounds of these training stimuli. These compounds comprised either elements that had elicited the same response (congruent trials) or different responses (incongruent trials) during training.

## Results

### Histological evaluation of the lesions

**Figure 2.**
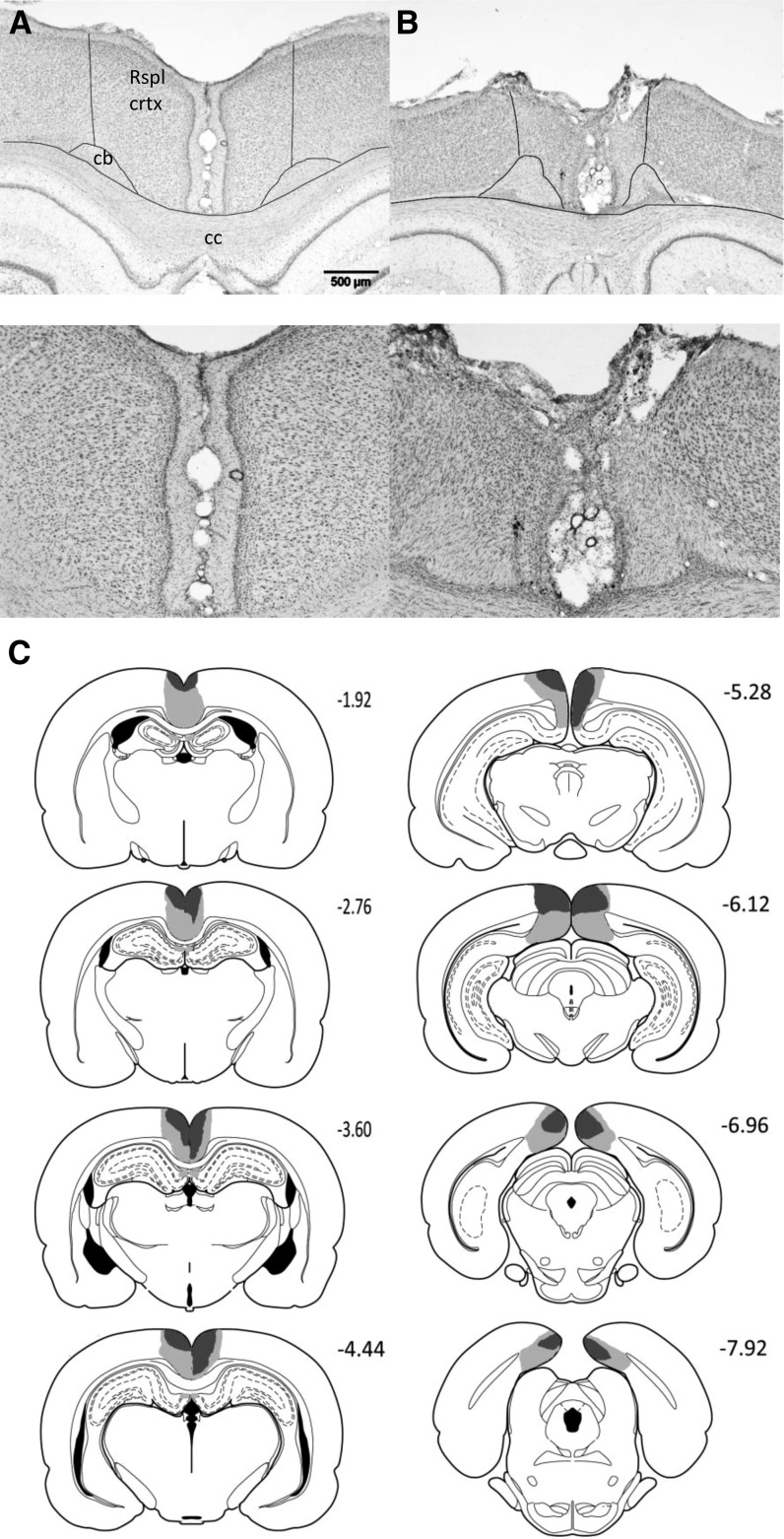
Location and extent of retrosplenial cortex lesions. Photomicrographs of a coronal section immunostained for Nissl from a sham control (*A*) and from a case with a mid-sized retrosplenial lesion (*B*). (*C*) Coronal reconstructions showing the case with the minimal (black) and the maximal (black and gray areas) extent of retrosplenial cortex damage. The numbers in *C* indicate the distance (in millimeters) from bregma (adapted from Paxinos and Watson 2007, with permission from Elsevier © 2007). Scale bar, 500 μm. (cc) Corpus callosum, (cb) cingulum bundle.

The retrosplenial cortex (RSC) surgeries (*n* = 16) consistently produced marked cell loss and extensive gliosis throughout almost the entire retrosplenial cortex (Fig. 2). Anterior to the splenium, the lesions were largely complete, except in two cases where there was some granular cortex sparing (one bilateral). The anterior cingulate cortex was spared in all animals. Caudal to the splenium there was partial sparing of granular retrosplenial cortex in five cases (three bilateral). Additional cell loss occurred in a discrete part of the most dorsal portion of CA1 in the septal hippocampus (one case bilateral, five unilateral). In nine cases (three bilateral), narrowing of the medial blade of the dentate gyrus was seen on just a few sections immediately caudal to the splenium. These same cases often showed very restricted cell loss in the dorsal subiculum at the same level. In one animal there was some bilateral thinning of the parietal cortex. For any subsequent group analyses in which the RSC group was found to be impaired, additional analysis was performed to compare the animals with damage confined to the retrosplenial cortex with those with additional damage to the hippocampal formation. There was a total of 12 Sham animals.

### Behavioral

#### Experiment 1a: response choice during stimulus conflict

##### Acquisition of conditional discriminations

**Figure 3.**
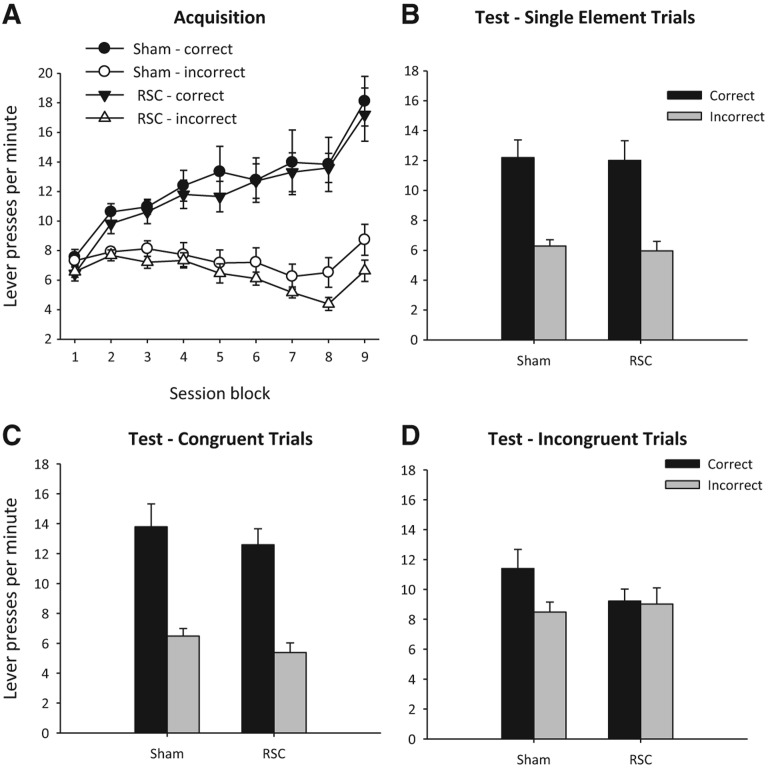
Experiment 1a: response conflict performance (correct and incorrect lever presses per minute, ±SEM). (*A*) Both groups showed accurate acquisition of the conditional discriminations. (*B*) In extinction, both groups showed accurate performance to the single elements used throughout acquisition. (*C*) In extinction, both groups showed accurate discrimination performance to congruent stimulus compounds. (*D*) For incongruent stimulus compounds, retrosplenial cortex lesions (RSC) abolished context-appropriate responding, in contrast to the Sham rats. (Congruent compounds consisted of stimulus elements that elicited the same response during training; incongruent compounds consisted of stimulus elements that elicited different responses during training.)

Animals acquired two instrumental discriminations, one visual and one auditory, in two distinct contexts and were rewarded for pressing the correct lever with different outcomes in each context (Fig. 1). Both Sham and RSC groups successfully acquired the visual and auditory conditional discrimination tasks, as reflected by their preference for the correct lever (*F*_(1,25)_ = 121.6, *P* < 0.001) (Fig. 3A) and lack of any lesion effect or interaction with session block (both *F* < 1). In contrast, there was an overall change in response levels across the blocks of training (*F*_(8,200)_ = 13.7, *P* < 0.001) as preference for the correct lever emerged with training (lever by session block interaction, *F*_(8,200)_ = 48.8, *P* < 0.001). One RSC animal failed to acquire the visual discrimination (no consistent preference for the correct lever across all nine blocks of training, and during the final block of training prior to test the rat pressed the incorrect lever more than the correct lever) and was, therefore, excluded from all behavioral analyses.

##### Extinction test performance

Animals underwent extinction test sessions in which compounds of the training stimuli were presented*.* These compounds combined stimulus elements that dictated either the same (“congruent”) or different (“incongruent”) instrumental responses during initial training (Fig. 1). The mean response rates (correct vs. incorrect) for each of the three trial types (single element, congruent, and incongruent) were analyzed after the four counterbalanced test sessions were combined. Thus, across the four extinction tests, there were 12 trials in total per trial type. As these tests were conducted in extinction, lever press behavior across the full 60 sec of each trial was analyzed.

1. Single stimulus elements. Both groups showed accurate conditional discrimination performance when given a single training stimulus during extinction (Fig. 3B), i.e., when tested on the stimulus elements acquired during training and when there was no conflict. Consequently, both groups produced more correct responses (*F*_(1,25)_ = 53.5, *P* < 0.001) with no effect of lesion or interaction (both *F* < 1).
2. Congruent compound stimuli. Again, both groups produced more correct than incorrect responses (Fig. 3C) during presentation of congruent compound stimuli (*F*_(1,25)_ = 103.9, *P* < 0.001), i.e., audiovisual compounds composed of single elements which during initial training had been associated with the same response (i.e., no conflict). There was no effect of lesion or interaction (both *F* < 1).
3. Incongruent compound stimuli. Incongruent trials consisted of audiovisual compounds of single elements that during training had elicited different responses in different contexts (Fig. 1). Consistent with previous reports (Haddon and Killcross 2006a; Haddon et al. 2008), Sham animals appeared to use contextual cues to disambiguate the conflicting response information, and so responded according to the stimulus element that had previously been trained in that same test context (Fig. 3D). In contrast, the RSC group pressed the context-appropriate and context-inappropriate levers at equivalent rates (Fig. 3D). This pattern is reflected in the lever by lesion interaction (*F*_(1,25)_ = 4.4, *P* < 0.05). In addition, there was a main effect of lever (*F*_(1,25)_ = 5.8, *P* < 0.05) but no lesion effect (*F* < 1). Simple effects analysis of the lever by lesion interaction revealed context-appropriate responding by the Sham (*F*_(1,25)_ = 9.2, *P* < 0.01) but not the RSC (*F* < 1) group. To verify that this deficit was specifically related to retrosplenial damage, follow-up statistical analyses were performed on the subset of animals with any damage that extended beyond the retrosplenial cortex. An ANOVA with between-subjects factors of subgroup and within-subjects factor of lever was conducted. This analysis revealed no effect of subgroup (*F* < 1) and no interaction between subgroup and incongruent test performance (*F* < 1).

#### Experiment 1b: selective reward devaluation on contextual conditioning

**Figure 4.**
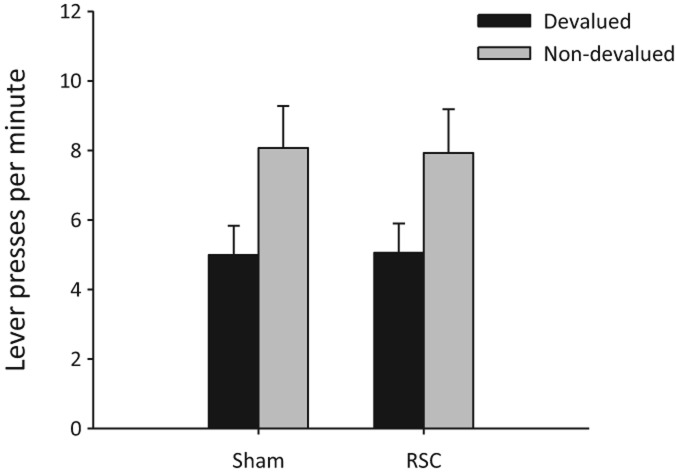
Experiment 1b: selective reward devaluation on contextual conditioning. Lever presses per minute (±SEM) after pre-feeding with the outcome associated with the test context (devalued condition) and after pre-feeding with the outcome not associated with the test context (nondevalued condition). Normal effect of outcome devaluation on responding to contextual cues in both Sham and RSC-lesioned animals.

To determine whether the animals were able to discriminate the two test contexts, Experiment 1b assessed the effect of reward devaluation on instrumental responding to contextual cues. Both groups (Fig. 4) displayed intact context–reward associations as they responded less during extinction when pre-fed the reward that had previously been earned in that test context compared to when pre-fed the reward associated with the other context (*F*_(1,25)_ = 12.9, *P* < 0.001). There was no effect of lesion or any interaction with lesion (both *F* < 1). During the prior reminder sessions, accurate conditional discrimination performance was observed in both groups (main effect of lever [*F*_(1,25)_ = 187.9, *P* < 0.001], other analyses [*F* < 1]).

#### Experiment 1c: selective reward devaluation on response choice during stimulus conflict

In order to probe the mechanisms underpinning response choice during stimulus conflict, rats were pre-fed to satiety with the outcome associated with the test context (same condition) or with the outcome associated with the alternative nontest context (different condition), and then responding to both congruent and incongruent compounds was assessed in extinction.

During the preliminary reminder training both groups correctly solved the conditional discriminations (*F*_(1,25)_ = 133.8, *P* < 0.001), with no lesion effect or interaction (maximum *F*_(1,25)_ = 2.7, *P* = 0.11). Preliminary analyses of the extinction sessions revealed no effect of test order or test context, so data were collapsed.

##### Congruent compound stimuli after pre-feeding with either the same or different context reward

**Figure 5.**
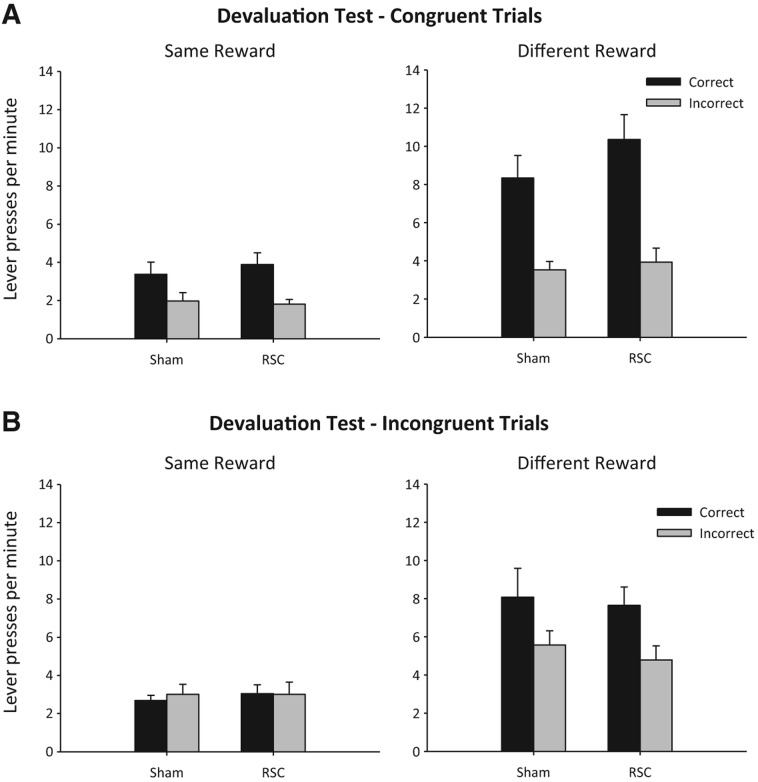
Experiment 1c: selective reward devaluation on response conflict. Correct and incorrect lever presses per minute (±SEM) for sham and retrosplenial cortex-lesioned animals to congruent stimulus compounds (*A*) and incongruent compounds (*B*). The graphs depict lever responding when tested in a context in which the devalued outcome had previously been earned (“same” reward condition, *left* column) or when the devalued outcome had not previously been earned in that test context (“different” reward condition, *right* column). Accurate performance to the congruent stimuli in both devaluation conditions was observed, but the overall rate of responding was reduced in the “same” reward condition (*A*). For incongruent trials (*B*), when tested in a context in which the devalued outcome had previously been earned (“same” reward condition), context-appropriate responding was abolished in both sham and retrosplenial cortex lesion animals. In contrast, when tested in a context in which the devalued outcome had not previously been earned (“different” reward condition [*B*]) both sham and retrosplenial cortex-lesioned animals responded in a context-appropriate manner.

There were no lesion effects or lesion interactions (all *F* < 1). As predicted, devaluation decreased responding when animals were pre-fed the “same” reward as associated with the test context condition, relative to when pre-fed the “different” reward, i.e., associated with the other context (*F*_(1,25)_ = 37.1, *P* < 0.001; “same” vs. “different” responding) (Fig. 5A). Animals also produced more correct than incorrect responses (*F*_(1,25)_ = 80.3, *P* < 0.001) (Fig. 5A). Although the magnitude of this effect was larger in the “different” condition (pre-feeding by lever interaction, *F*_(1,25)_ = 16.3, *P* < 0.001) (Fig. 5A), nonetheless, the rats displayed the correct pattern of responding in both the “same” (*F*_(1,25)_ = 30.5, *P* < 0.001) and “different” (*F*_(1,25)_ = 47.5, *P* < 0.001) conditions. None of the trends was affected by lesion as there were no effects of lesion or interaction with lesion (max *F*_(1,25)_ = 1.9, *P* = 0.18).

##### Incongruent compound stimuli after pre-feeding with either the same or different context reward

Pre-feeding the reward associated with the test context reduced response rates relative to when animals were pre-fed the reward associated with the alternative context (*F*_(1,25)_ = 27.9, *P* < 0.001; “same” vs. “different”) (Fig. 5B). Furthermore, pre-feeding the reward associated with the alternative context (“different” condition) caused both groups to produce more correct (context-appropriate) than incorrect (context-inappropriate) responses (Pre-Feeding by Lever interaction, *F*_(1,25)_ = 8.9, *P* < 0.01) (Fig. 5B). Conversely, pre-feeding the contextually appropriate reward (“same” condition) reduced the ability of the context to promote responding toward the correct lever as rats now distributed their responding equally between the two levers (no effect of Lever, *F* < 1) (Fig. 5B). These trends were not influenced by surgery group as there were no effects or interactions with lesion (all *F* < 1).

## Discussion

Conflict control was taxed by simultaneously presenting pairs of stimuli that had individually elicited opposing (“incongruent”) responses due to their initial conditional discrimination training in different contexts (Experiment 1a). Consistent with previous reports (Haddon and Killcross 2006a, 2008), sham-operated animals relied on contextual information to resolve this conflict task. Although rats with retrosplenial cortex lesions successfully learnt the initial conditional tasks, these animals failed to use task-setting cues provided by the contexts to help select between the context-appropriate and context-inappropriate levers during incongruent trials. Although it might be supposed that this abnormality stems from a failure to discriminate the contexts (Bar and Aminoff 2003; Robinson et al. 2012) that interpretation can be excluded given our other experimental results. Consequently, this study reveals an important contribution from the retrosplenial cortex to cognitive control when under particular conflict conditions.

The failure of the RSC-lesioned animals to use contextual task-setting cues to disambiguate the response conflict during incongruent compound presentation is most unlikely to be due to impaired learning of the stimulus–response associations required to perform this task successfully. First, the RSC group acquired the initial conditional discriminations at the same rate and with the same accuracy as the sham animals. Moreover, no lesion differences were found during either single element trials or compound congruent trials in the extinction tests, confirming accurate conditional discrimination performance in both groups. That the impairment was not simply a failure to discriminate contexts can also be eliminated. In Experiment 1b we clearly demonstrated that the retrosplenial lesion group was able to discriminate the two contexts as these animals reduced responding in a context-specific manner when selectively sated on the reward associated with that test context. Thus, despite prior evidence that lesions to the retrosplenial cortex can disrupt contextual fear conditioning, which requires rapid learning about contexts (e.g., Keene and Bucci 2008a; Robinson et al. 2012; but see Lukoyanov and Lukoyanova 2006), the RSC group was able to associate a specific context with a specific reward. The conclusion is that retrosplenial lesions impair how that contextual information is used rather than generally disrupt the learning of contextual information or impair all inhibition processes.

In the current task, the context not only comprised the two distinct experimental chambers but also the different rewards earned in each context as well as time of day. Thus there were potentially several sources of contextual information available to the animals. During discrimination training, rats were required to form associations between specific stimuli and responses but could readily perform the conditional discrimination task by learning these associations without drawing on contextual information. An inability to link contextual cues with the various cues and responses would similarly leave both single element and congruent test performance intact, as performance on these trials is not reliant on the use of contextual information. Conversely, a failure to link these different cue and response associations with contextual information and to form a combined or configural representation of these various elements could underlie the retrosplenial lesion group's selective impairment during incongruent trials, when animals were required to utilize contextual information to resolve response conflict. However, the results of Experiment 1c make this an unlikely explanation of the pattern of results obtained. In Experiment 1c, we used selective reinforcer devaluation to examine the nature of the representations underpinning task performance. If the RSC group had failed to link contextual information with specific cues and responses, then it is unlikely that the effect of a change in reward value on test performance would be contextually specific. However, both groups markedly reduced their overall rates of responding when pre-fed the reward associated with that test context (“same” condition) relative to when pre-fed the reward associated with the alternative context (“different” condition). Thus, the RSC rats could link an outcome to a specific context and its associated cues and responses.

Furthermore, the results from the incongruent trials of Experiment 1c show that the RSC lesion animals were able, under specific circumstances, to use contextual cues to resolve the conflict engendered by presentation of the incongruent compounds. Pre-feeding to satiety, the outcome associated with the context-inappropriate cues and responses (different condition, Experiment 1c) appeared to restore accurate incongruent performance in the RSC rats, as these animals were now able to inhibit context-inappropriate responses and show an apparently normal response bias to the cue trained in the test context (context-appropriate responding). Thus, reducing the motivational significance of the outcome associated with the competing cues/responses appeared to diminish the influence of this conflicting information on current behavior and allowed the RSC group to use contextual information to disambiguate the conflicting cues and respond in a context-appropriate manner. Conversely, when sated on the reward associated with the test context (“same” condition, Experiment 1c), neither group resolved the response conflict. This pattern, as found in other studies, suggests that devaluing the motivational significance of the context-appropriate cues attenuated the ability of these cues to elicit the correct lever responses (Haddon and Killcross 2006b, 2007). Taken together, the findings from Experiment 1c demonstrate that a failure to integrate contextual, cue, and response information cannot provide a complete account of the selective impairment on incongruent trials observed in Experiment 1c. Rather, these results highlight how the different rewards, as well as other sources of contextual information, help guide the conflict responses, and that this motivational component, rather than an inability to use contextual information, contributed to the lesion-induced deficit seen in Experiment 1a. Attenuating the influence of this conflicting information allowed the retrosplenial lesion animals to perform the task at comparable levels to the sham group. Recent work in humans has similarly highlighted the importance of reward-related processes in both guiding and impeding conflict processing in tasks such as the Stroop (e.g., Krebs et al. 2010, 2011).

The proposal that the retrosplenial cortex helps select task-setting cues for the production of appropriate responses is supported by findings from studies of rodent spatial learning and navigation (Aggleton 2010). In radial arm-maze studies, it is often only when intra- and extramaze cues are opposed that consistent retrosplenial lesion impairments emerge (Vann et al. 2003; Vann and Aggleton 2004; Pothuizen et al. 2008). Similarly, the retrosplenial cortex appears to be important when animals select between relevant and irrelevant spatial information (Wesierska et al. 2009) and when there is a shift from light to dark conditions during testing (Cooper and Mizumori 1999). These findings accord with the more general notion that the retrosplenial cortex has a role in switching between representations based on different spatial metrics (Byrne et al. 2007; Vann et al. 2009). Some additional support for the involvement of the retrosplenial cortex in stimulus evaluation and selection comes from nonspatial tasks; for example, electrolytic retrosplenial cortex lesions disrupt feature-negative discriminations (Keene and Bucci 2008b; Robinson et al. 2011), consistent with a role for the retrosplenial cortex in cue competition. Moreover, retrosplenial cortex neurons may encode behaviorally significant cues that predict rewards, or elicit a behavioral response or its omission (e.g., Gabriel et al. 1989; Smith et al. 2002). These neurons have been shown to respond selectively to contextual cues that are necessary for the retrieval and execution of task-appropriate responding (e.g., Smith et al. 2004, 2012).

The retrosplenial cortex is interlinked with frontal sites already strongly implicated in cognitive control. For example, studies of Stroop performance in humans consistently highlight activity in frontal areas (e.g., MacDonald et al. 2000; Carter and van Veen 2007). Within the frontal region, the anterior cingulate cortex appears necessary for normal conflict detection and task-relevant action selection (e.g., Carter et al. 1998, Botvinick et al. 1999; Dias and Aggleton 2000; Rushworth et al. 2004; de Wit et al. 2006). Consistent with the suggestion that the anterior cingulate is required for conflict detection, Haddon and Killcross (2006a) found that anterior cingulate lesions disrupt incongruent trial performance on the current task, but only during the first 10 sec of stimulus presentation. Rats with lesions in the medial prefrontal (prelimbic) cortex (Haddon and Killcross 2006a), another site repeatedly linked with rodent cognitive control (Ragozzino et al. 1999; Dias and Aggleton 2000), also abolish the contextual control of response conflict performance. Despite the severity of the impairment on the Stroop, there is no reason to suppose that retrosplenial lesions result in a global loss of inhibition (e.g., Aggleton et al. 1995) and that the effects, like those of damage to different frontal regions, are more confined to specific aspects of cognitive control.

The present findings, therefore, extend the functional network underlying cognitive control and may help to explain the occasional reports of posterior cingulate activations during Stroop Task performance (e.g., Peterson et al. 1999; Badzakova-Trajkov et al. 2009). Furthermore, there is evidence that this control network is not simply defined by its prefrontal connectivity. This conclusion derives from the finding that rat hippocampal lesions can enhance, rather than disrupt, incongruent-trial performance on the present Stroop-based task (Haddon and Killcross 2007), despite the hippocampus being directly interconnected with both prelimbic and retrosplenial cortices. This double dissociation reveals a transformation of function across directly interconnected sites. Consistent with these results, Stroop performance is also unimpaired in patients with hippocampal damage (e.g., Parslow et al. 2005). Although it has long been appreciated that the posterior cingulate region serves as a potential site for the integration and evaluation of diverse sensory information (Vogt et al. 1992), the present findings reveal a specific role for the retrosplenial cortex in situations of response conflict.

## Materials and Methods

### Subjects

Subjects were 28 male rats (Lister-Hooded strain, Harlan Bicester, UK) housed in pairs under diurnal light conditions (14-h light/10-h dark). Large cardboard tubes and wooden chewsticks were provided in the home cages. Behavioral testing occurred during the light phase. Water was available ad libitum. All experiments were conducted in accordance with the UK Animals (Scientific Procedures) Act of 1986 and related guidelines.

### Surgery

Rats were randomly assigned for either retrosplenial cortex (“RSC,” *n* = 16) or sham (“Sham,” *n* = 12) surgeries. All animals (mean weight 278 ± 5.5 g) were first injected with atropine (0.03 mL of a 600 µg/mL solution, intraperitoneal [i.p.], Matindale Pharma) and then deeply anesthetized with 6% sodium pentobarbital (i.p., 60 mg/kg, Sigma) dissolved in sterile saline and alcohol. Anesthesia was maintained with isoflurane (0.5% in O_2_). The remainder of the surgical procedures closely followed those previously described (Pothuizen et al. 2008). The retrosplenial lesions were made by injecting 0.09 M *N*-methyl-D-aspartic acid (NDMA, Sigma Aldrich) in seven sites per hemisphere. The anterior–posterior (AP) injection coordinates were taken from bregma, the mediolateral (ML) coordinates from the sagittal sinus, and the dorsoventral (DV) coordinates from dura. The stereotaxic coordinates and injection volumes were:

1. 0.27 µL at −1.8 (AP), ±0.4 (ML), −1.0 (DV);
2. 0.27 µL at −2.8 (AP), ±0.4 (ML), −1.1 (DV);
3. 0.27 µL at −4.0 (AP), ±0.4 (ML), −1.1 (DV);
4. 0.29 µL at −5.3 (AP), ±0.4 (ML), −2.4 (DV);
5. 0.29 µL at −5.3 (AP), ±0.9 (ML), −1.4 (DV);
6. 0.29 µL at −5.3 (AP), ±0.9 (ML), −1.8 (DV);
7. 0.1 µL at −7.5 (AP), ±1.0 (ML), −1.1 (DV).

The surgical shams received the identical procedure except that the needle was not lowered into the cortex and no NDMA infusions were made. All rats recovered well following surgery. After a minimum of 10 d of post-operative recovery, rats were gradually reduced to 85% of their free-feeding weights.

### Apparatus

Eight operant chambers (30 cm wide × 24 cm deep × 21 cm high, Med Associates) were used. Each chamber had three aluminum walls, with a Perspex door serving as the fourth wall. In four “white” chambers, the walls and ceilings were lined with white paper with a single 5-cm black stripe, fixed behind transparent Perspex. The other four chambers were “plain” aluminum (Fig. 1). Each chamber floor consisted of 19 stainless-steel rods (3.8 mm in diameter, spaced 1.6 cm apart). In four chambers the sawdust beneath the floor was mixed with cumin powder, in the other four it was mixed with paprika powder. Each chamber was illuminated by a 3-W houselight located at the top center of the left wall. Food pellets (45 mg, Noyes) could be delivered into a recessed magazine located in the center of the right chamber wall. Fifteen percent sucrose solution could be delivered via a dipper into the same magazine. Two flat-panel retractable levers were located to the left and right of the magazine. Auditory stimuli consisted of a 2-kHZ tone and 10-Hz train of clicks, both delivered through ceiling speakers. Visual stimuli consisted of either two “flashing” (0.1 sec on, 0.1 sec off) panel lights (each 2-cm diameter, located above the retractable levers) or two “steady” panel lights plus illumination of the magazine light.

### Experiment 1a: response choice during stimulus conflict

#### Behavioral training

##### Lever press training

After four training sessions, each rat would lever press for a single food pellet or 0.1 mL of the sucrose solution on a random interval schedule (RI15) such that once in every 15 sec, on average, a reward became available following a lever press.

##### Conditional discrimination training

Next, rats received 18 d on two concurrent conditional discriminations (Fig. 1). There were two sessions a day, one in each of the two contexts (e.g., white/cumin and plain/paprika). One session was conducted in the morning and the other in the afternoon (minimum of 4 h between each session). Correct responses were rewarded with food pellets in one context and sucrose solution in the other.

In one context (e.g., white chamber) rats were presented with visual cues (flashing or steady lights). During one visual stimulus (e.g., steady lights), only responding on the left lever was reinforced; during the other visual stimulus (e.g., flashing lights), only responding on the right lever was reinforced (Fig. 1). In the other context (e.g., plain chamber), auditory stimuli were used (click or tone). For one auditory stimulus (e.g., click) only responding on the left lever was reinforced, while for the other auditory stimulus (e.g., tone) only responding on the right lever was reinforced (Fig. 1).

The contexts, stimuli, responses, and rewards were counterbalanced across animals as far as possible, ensuring that each group experienced both of the discriminations (auditory and visual) in both contexts (spot and check) with both rewards (sucrose and pellets). We also made sure that each group experienced all of the possible cue–lever combinations. Each session contained 24 trials. In one context, a session comprised 12 tones and 12 clicks. In the second context, a session comprised 12 steady and 12 flashing lights. There was a mean inter-stimulus interval of 60 sec (range 30–90 sec). Both levers were present during each stimulus presentation and retracted during the inter-stimulus interval. Each stimulus presentation lasted 60 sec. During the first 10 sec of each trial, reinforcement was unavailable so that discrimination performance was uncontaminated by reinforcement. During the remaining 50 sec, reinforcement was available on the RI15 schedule of reinforcement (see above).

##### Extinction sessions

All rats next underwent four extinction sessions: two in each of the two training contexts (Fig. 1). The animals first received 2 d of extinction testing (one in each context) and then, after 2 d of reminder training on the original conditional discriminations, two more extinction sessions. The test order (Context 1 vs. Context 2) was counterbalanced across animals. Extinction testing consisted of presenting either individual training stimuli (“single element”) or audiovisual compounds of the training stimuli (“congruent” and “incongruent”) (see Fig. 1). Rats received 18 extinction trials, three blocks of each trial type (three single element, three congruent, and three incongruent) and for each trial type there were two possible stimuli or stimulus compounds. Trial order was block randomized, with each stimulus or compound being presented once in each block of six trials. Both levers were available but responding was not reinforced. Stimulus duration was 60 sec and there was a mean inter-stimulus interval of 60 sec.

Congruent stimulus compounds consisted of visual and audio elements that had been trained to elicit the same lever response in both contexts. For example, if both click and steady light had signaled a rewarded left lever press, when presented together both stimuli should elicit the same lever response irrespective of context (Fig. 1). In contrast, incongruent stimulus compounds comprised individual elements that, after training, elicited different responses. For example, within the incongruent compound “flashing light + click,” the flashing light elicited a right lever press in Context 1 but the click elicited a left lever press in Context 2 (Fig. 1).

### Experiment 1b: selective reward devaluation on contextual conditioning

By pre-feeding one reward to satiety, normal rats should show a selective reduction in responding for the sated reward associated with that particular context. This response inhibition was tested after 2 d of reminder training on the original conditional discriminations. The rats then received two 15-min extinction sessions during which the houselight was illuminated and both levers available. Prior to each extinction session, the animals were put in an adjacent room where they received 30-min free access to one of the two rewards (15% sucrose solution or the food pellets) earned during training. Each rat was tested twice in the same context (counterbalanced so that half were tested in Context 1 and half in Context 2), with testing order (devalued vs. nondevalued) also counterbalanced.

### Experiment 1c: selective reward devaluation on response choice during stimulus conflict

This experiment further examined contextual control. Each stimulus element and context in Experiment 1a is associated with a specific reward (pellet or sucrose), thus the motivational salience of the different rewards may well contribute to behavior in response conflict (“incongruent”) trials. Indeed, context-appropriate responding during incongruent trials can be selectively abolished by devaluing the reward associated with the context in which the test occurs (Haddon and Killcross 2006b, 2007).

All animals first received 2 d of reminder training on the original conditional discrimination. Each animal was then tested in extinction four times, twice in each context, interspersed by two more days of reminder training. Before each extinction test, the animals were pre-fed (see Experiment 1b) either the reward associated with that context (“same”) or the reward associated with the alternative context (“different”). Testing order (context and reward) was fully counterbalanced. Unlike Experiment 1a, each session consisted of only congruent and incongruent trials, with three blocks of each trial type.

### Histology

Histological procedures included the staining of coronal sections for Nissl substance. At the end of behavioral testing, the rats were deeply anesthetized with sodium pentobarbital (60 mg/kg, i.p, Euthatal, Merial Animal Health) and then transcardially perfused with 0.1 M phosphate-buffered saline (PBS) at room temperature for ∼2 min (flow rate 35 mL/min), followed by a 4% solution of depolymerized paraformaldehyde in 0.1 M phosphate buffer for ∼10 min at a flow rate of 35 mL/min. The brains were removed and post-fixed for 4 h in the same fixative and then cyroprotected in 25% sucrose solution (in PBS) overnight. Four adjacent series of coronal sections (40 µm) were cut on a freezing sliding microtome. Three series were collected and stored in cyroprotectant for subsequent processing. One in four series was directly mounted onto gelatin-coated slides and, when dry, stained with cresyl violet, a Nissl stain. The sections were then dehydrated through an alcohol series, cleared with xylene, and cover-slipped with the mounting medium DPX.

### Data analysis

Performance on the conditional discrimination training and reminder sessions, as well as the test sessions, was calculated as a rate of lever presses per minute on both the correct and the incorrect levers. For incongruent test trials, responding according to the element that had previously been trained in that test context (i.e., context-appropriate) was deemed a correct response, while responding according to the element that had previously been trained in the alternative context (i.e., context-inappropriate) was deemed an incorrect response. For the conditional discrimination task, rates were calculated using only the first 10 sec of stimulus presentation (during which no reinforcement was available). For the extinction test sessions, rates were calculated for the entire stimulus presentation (60 sec). For the conditional discrimination training, an ANOVA with a between-subjects factor of Group (Sham or RSC) and within-subject factors of Lever (correct and incorrect) and Session (nine blocks of two sessions) was conducted. ANOVAs with a between-subjects factor of Group (Sham and RSC) and Lever (correct and incorrect) were carried out separately on each trial type (single-element, congruent, and incongruent compounds). In Experiment 1c, there was an additional within-subject factor of pre-feeding (either the reward associated with the test context [“same”] or the reward associated with the alternative context [“different”]). Where appropriate, interactions were explored with simple effects analysis based on the pooled error term. The α level was set at *P* < 0.05 for all comparisons.
